# The efficacy of topical tranexamic acid in total hip arthroplasty: a meta-analysis

**DOI:** 10.1186/s12891-016-0923-0

**Published:** 2016-02-16

**Authors:** Shubiao Chen, Kezhou Wu, Gengbin Kong, Weili Feng, Zhihua Deng, Hu Wang

**Affiliations:** Department of Orthopaedics, First Affiliated Hospital of Shantou University Medical College, Shantou, Guangdong 515041 China

**Keywords:** Total hip arthroplasty, Tranexamic acid, Topical, Blood loss

## Abstract

**Background:**

Topical tranexamic acid (TXA) has been shown to be effective in reducing blood loss and the need for transfusion after total knee arthroplasty. However, the effectiveness of topical TXA use in total hip arthroplasty (THA) still remains unclear. The purpose of this meta-analysis is to examine the safety and efficacy of topical use of TXA following THA.

Hypothesis: Topical TXA reduces blood loss and transfusion rates without increasing risk of deep vein thrombosis in patients with THA.

**Methods:**

An electronic literature search of PubMed, Embase, the Cochrane Library, Web of Science and Chinese Biomedical Database was performed, to identify studies published before February 2015. All randomized controlled trials and cohort studies evaluating the efficacy of topical TXA during THA were included. Two independent authors identified the eligible studies, assessed their methodological quality, and extracted data. The data were using fixed-effects or random-effects models with (standard) mean differences and risk ratios for continuous and dichotomous variables, respectively. Data were analysed using RevMan 5.3 software.

**Results:**

Fourteen studies encompassing 2594 patients met the inclusion criteria for our meta-analysis. Our meta-analysis indicated that when compared with the placebo group, topical use of TXA significantly reduced total blood loss (MD = −297.65 ml, 95 % CI −371.68 ml, 116.08 ml; *P* < 0.01), drainage loss (MD = −164.68 ml, 95 % CI −236.63 ml, −92.73 ml; *P* < 0.01), transfusion rate (RR = 0.26, 95 % CI 0.17, 0.40; *P* < 0.01) and with less of a drop in haemoglobin level (SMD = −0.66, 95 % CI −0.91, −0.41; *P* < 0.01) after primary THA. No significant difference in length of hospital stay (MD = −0.40, 95 % CI −0.91, 0.11; *P* = 0.14), deep vein thrombosis (RR = 1.19, 95 % CI 0.40, 3.57; *P* = 0.16) and pulmonary embolism (RR = 1.11, 95 % CI 0.11, 10.81; *P* = 0.21) among the study groups.

**Conclusions:**

Topical TXA could significantly reduce total blood loss, drainage loss, transfusion rates and decrease haemoglobin level following THA, without increasing risk of venous thromboembolisms.

## Background

Total hip arthroplasty (THA) is one of the most successful procedures in orthopaedic elective surgery for end-stage arthritis diseases of hip [1]. It is estimated that by 2030, the demand for primary THA will grow by 174 % to 572,000 [2]. However, THA is associated with substantial blood loss, ranging from 1188 to 1651 ml [3] and the transfusion rate is 16 to 37 %, which often resulting in postoperative blood transfusions subsequently [4, 5]. Perioperative transfusions are associated with risks and complications, including transmission of infectious agents, hemolytic transfusion reaction, and short-term mortality [6, 7].

A number of techniques are currently used to decrease the need for postoperative blood transfusions, including autologous blood transfusion, intraoperative blood saving, local anesthesia, hypotensive anesthesia and antifibrinolytic drugs including, tranexamic acid (TXA) administration [8]. TXA is a synthetic derivative of the amino acid lysine which decreases fibrinolysis activity by competitively inhibiting lysine binding sites on plasminogen molecules [9]. TXA enables the body to retain blood clots more effectively, thereby reduce bleeding. Numerous studies have investigated the effectiveness of intravenous TXA in THA [10, 11] and meta-analyses have revealed that intravenous TXA could reduce the blood loss and transfusion rates following THA, without increasing thromboembolic complications [12, 13].

Recently, topical use of TXA has been popular and has been shown to be effective in lowering blood loss in total knee arthroplasty. However, there are still controversy about the efficacy of TXA in THA. Some studies reported better outcomes in TXA for reducing blood loss [14–23] while other studies found no differences for transfusion rates [24–27] between the two groups.

Accordingly, we systematically reviewed the current evidence to investigate whether topical TXA was effective for reducing blood loss and transfusion rates following THA. The hypothesis of our study was that topical TXA could reduce blood loss and transfusion rates without increasing the risk of deep vein thrombosis after TKA postoperatively.

## Methods

We conducted this study according to the methods of the Cochrane Handbook for Systematic Reviews of Interventions [28]. This systematic review and meta-analysis was reported according to the Preferred Reporting Items for Systematic Reviews and Meta-Analyses (PRISMA) Statement [29].

### Search strategy

We identified comparative studies comparing TXA with placebo by searching Embase, PubMed, Cochrane Library, Web of Knowledge and Chinese Biomedical Database since the inception. We used the following combined text and MeSH terms: “Tranexamic acid”, “topical”, “local”, “intra articular”, “hip arthroplasty” and “hip replacement”. There were no language restrictions. We also checked the bibliographies of identified reports and relevant review articles for other potentially relevant studies.

### Study selection and inclusion criteria

Two authors independently conducted the initial search, deleted duplicate records, screened the titles and abstracts for relevance and identified papers. Study selection was performed according to the following inclusive criteria: (1) randomized controlled trials (RCTs) or cohort studies (CSs); (2) participants underwent primary THA; (3) interventions including topical (intra-articular) and IV-TXA; and (4) reported outcomes, including postoperative total drain output, total blood loss, maximum postoperative Hb drop, blood units transfused per patient, the number of patients receiving blood transfusion, the incidence of DVT and PE. Studies with cadaver and artificial models and patients with bleeding disorders were excluded.

### Data extraction

Two investigators independently extracted data, with disagreement resolved in consultation with another investigator. For each article data on the following study characteristics were collected if available: geographical location, year of publication, age of participants, proportion of female/male participants, intervention and length of follow-up.

The primary outcome was total blood loss and total drain loss. The second outcome included transfusion rates, hemoglobin drop, length of hospital stay and complications (deep venous thrombosis, pulmonary embolism and infection).

### Quality assessment and grading the quality of evidence

Two reviewers independently assessed the risk of bias of individual studies according to the Oxford Centre for Evidence-based Medicine Levels of Evidence [30]. The level of each trial was classified as A, B, C, or D. Disagreements on the inclusion of studies were resolved by the senior reviewer until a consensus was reached.

To assess the level of evidence for an intervention, the Grading of Recommendations, Assessment, Development and Evaluation (GRADE) was used. Quality of evidence was classified as high, moderate, low, or very low based on the judgements for the outcome regarding risk of bias, inconsistency, indirectness, imprecision, and other considerations (publication bias). Summary tables were constructed with GRADEpro version 3.6.

### Statistical analysis

The results of meta-analysis were analysed using RevMan 5.3. Dichotomous were calculated as the odds ratios (OR). Continuous data were expressed as the mean difference (MD) or as the standardised MD (SMD) depending on the similarity of the used scales. To measure heterogeneity among all studies, we used I^2^ statistic. If I^2^ is less than 50 %, it represented low heterogeneity and Fixed-effects models were used. The outcomes were pooled using random-effects models If I^2^ more than 50 %. In case of heterogeneity, we planned a subgroup to explore possible differences.

## Results

### Search results

**Fig. 1:**
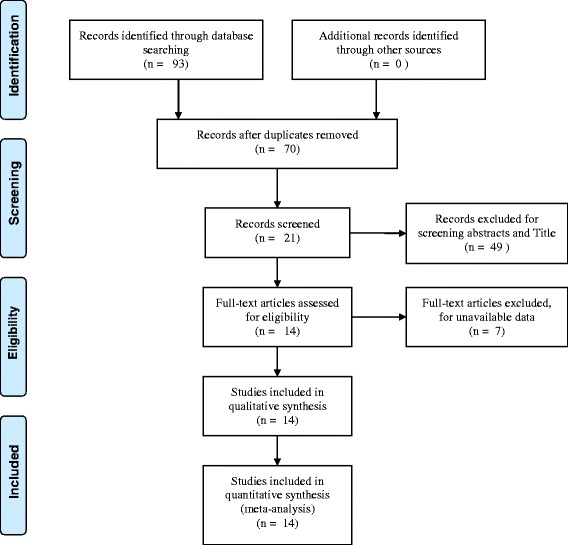
The flow chart of literature screening

The literature searches identified 93 publications that potentially met our criteria. After the initial screen of titles and abstracts, 21 articles remained for further investigation. After detailed assessments, there were 14 eligible studies [14–27] (one published as abstract only) (Fig. 1).

All included studies were published since 2013 and were reported in English except for three were in Chinese. The size of the TXA treatment and placebo groups ranged from 25 to 154 and 25 to 1047, respectively. The dose of topical TXA varied from 0.5 to 5 g. All included studies reported the transfusion trigger, of which, the level of six studies was less than 70, three less than 80, one less than 90, two less than 100. For DVT prophylaxis, five studies used low molecular weight heparin (LMWH), two physical methods, one used warfarin or aspirin while two studies did not report using any DVT prophylaxis. In our analysis, seven RCTs and seven nRCTs were identified for meta-analysis. Of which, seven studies were classified as A, six studies was B while one study was C. Basic demographic details and quality assessment of the included studies are listed in Table 1.

**Table 1:** The characteristics of included studies

Study (year)	Study type	No. IA vs IV	Age: IA vs IV	TXA group	Transfusion criteria	Thromboprophylaxis	Level of evidence
Alshryda 2013 [22]	RCT	80/81	66/63	1 g	Hb <70 g/l	LMWH	A
BagsBy 2014 [24]	nRCT	91/90	61.4/61.9	1 g	Hb <70 g/l	Warfarin/Asprin	B
Chang 2014 [14]	nRCT	154/234	57.2/56.7	3 g	Hb <100 g/l	--	B
Ding 2014 [23]	nRCT	30/30	60/61	3 g	Hb <80 g/l	--	B
Fan 2014 [15]	RCT	55/44	66.0/66.5	2 g	Hb <70 g/l	Physical	A
Gilbody 2014 [16]	nRCT	86/88	64/65	3 g	Hb <80 g/l	enoxaparin	B
Konig G 2013 [17]	nRCT	91/40	60/59	3 g	Hb <80 g/l	--	B
Machin 2014 [26]	nRCT	50/100	61/67.5	0.5 g	Hb <80 g/l	dabigatran	B
Martin JG 2014 [25]	RCT	25/25	63.9/63.0	2 g	Hb <70 g/l	Physical	A
Van Elst CE 2013 [18]	RCT	30/30	67.1/66.5	3 g	Hb <100 g/l	LMWH	A
Wei 2014 [19]	RCT	102/100	60.2/63.9	3 g	Hb <90 g/l	LMWH	A
Wind 2014 [27]	nRCT	70/1047	65/60	--	Hb <80 g/l	--	C
Yin 2014 [20]	RCT	30/30	50/53	2 g	Hb < 70 g/l	LMWH	A
Yue C 2014 [21]	RCT	52/49	60.9/63.7	3 g	Hb <70 g/l	LWMH	A

### Intervention

#### Total blood loss

The data of total blood loss was reported in ten studies [14, 16–23, 26]. The pooled result revealed that topical TXA significantly reduced total blood loss more than placebo (MD = −297.65 ml, 95 % CI −371.68 ml, 116.08 ml; *P* < 0.01). Nevertheless, these results should be interpreted with caution due to the presence of significant statistical heterogeneity (I^2^ = 71 %).

#### Total drain out

Six studies [14, 15, 20–23] including a total of 791 patients (363 topical TXA, 428 placebo) reported data regarding the total drain out. The analysis showed there was a significantly statistical difference between topical TXA and placebo group (MD = −164.68 ml, 95 % CI −236.63 ml, −92.73 ml; *P* < 0.01). This finding, however, should be interpreted with caution for significant statistical heterogeneity (I^2^ = 96 %).

### Transfusion rates

The data of transfusion rates was provided in 12 studies [14, 16, 17, 19–25, 27] involving 2764 patients. Meta-analysis demonstrated that topical TXA decreased the risk of transfusion rates more than placebo (OR = 0.26, 95 % CI 0.17, 0.40; *P* < 0.01). There was little statistical heterogeneity (I^2^ = 41 %).

### Hemoglobin drop

The result of hemoglobin drop was assessed in 11 studies [14–18, 20, 22–26] involving 1596 patients. Meta-analysis showed that there was significant difference in hemoglobin drop between topical TXA and placebo groups (SMD = −0.66, 95 % CI −0.91, −0.41; *P* < 0.01). There was significant heterogeneity among studies (I^2^ = 81 %).

### Length of hospital stay

Six studies [14, 16, 17, 19, 22, 26] with (1144 patients) reported details regarding length of hospital stay. The analysis showed there was no significant difference in length of hospital stay between topical TXA and placebo group (MD = −0.40, 95 % CI −0.91, 0.11; *P* = 0.006) in the presence of statistical heterogeneity (I^2^ = 83 %).

### Complications

The details of complication including DVT and PE was available in six studies [14, 20–22, 26, 27]. Meta-analysis demonstrated that there were no statistical differences in DVT (OR = 1.19, 95 % CI 0.40, 3.57; *P* = 0.006) and PE (OR = 1.11, 95 % CI 0.11, 10.81; *P* = 0.006) between the two groups in the absence of statistical heterogeneity (I^2^ = 0 %).

### Subgroup analysis

There was significant heterogeneity in the study outcomes. Therefore, subgroup analysis was performed based on the study type. There was no significant difference in all analyses between RCTs and nRCTs except total drain out. Table 2 shows the results of the meta-analysis and subgroup analysis.

**Table 2:** The results of meta-analysis and subgroup analysis

Outcomes	No. studies	No. Patients		SMD or OR (95 % CI)	*P* value	Heterogeneity
		IA TXA	IV TXA			
Total blood loss	10	642	635	−297.65 (−371.60 to −223.69)	<0.01	I^2^ = 71 %
RCT	5	281	247	−378.96 (−454.12 to −303.81)	<0.01	I^2^ = 18 %
nRCT	5	102	101	−218.88 (−271.68 to −166.08)	<0.01	I^2^ = 30 %
Total drain out	6	363	428	−164.68 (−236.63 to −92.73)	<0.01	I^2^ = 96 %
RCT	4	179	164	−187.97 (−276.77 to −99.18)	<0.01	I^2^ = 93 %
nRCT	2	184	284	−121.95 (−318.92 to 75.03)	0.22	I^2^ = 99 %
Transfusion rates	12	871	1893	0.29 (0.19 to 0.44)	<0.01	I^2^ = 46 %
RCT	5	319	304	0.21 (0.13 to 0.34)	<0.01	I^2^ = 0 %
nRCT	7	552	1589	0.36 (0.19 to 0.66)	<0.01	I^2^ = 59 %
Hb drop	11	774	822	−0.66 (−0.91 to −0.41)	<0.01	I^2^ = 81 %
RCT	5	292	280	−0.72 (−1.24 to −0.19)	<0.01	I^2^ = 88 %
nRCT	6	482	542	−0.61 (−0.87 to −0.36)	<0.01	I^2^ = 71 %
Length of hospital stay	6	542	602	−0.40 (−0.91 to 0.11)	0.13	I^2^ = 83 %
RCT	2	181	180	−0.27 (−1.29 to 0.75)	0.60	I^2^ = 66 %
nRCT	4	361	422	−0.51 (−1.37 to 0.35)	0.24	I^2^ = 88 %
DVTs	6	348	1355	1.19 (0.40 to 3.57)	0.75	I^2^ = 0 %
RCT	3	162	160	1.28 (0.31 to 5.29)	0.73	I^2^ = 0 %
nRCT	3	186	1195	1.07 (0.19 to 6.12)	0.94	I^2^ = 0 %
PE	2	224	1281	1.11 (0.11 to 10.81)	0.93	I^2^ = 6 %
nRCT	2	224	1281	1.11 (0.11 to 10.81)	0.93	I^2^ = 6 %

*Hb* hemoglobin, *DVT* deep venous thrombosis, *PE* pulmonary embolism

## Discussion

Based on 14 studies involving patients, the most important findings of this meta-analysis were that compared with placebo, topical TXA significantly reduced blood loss, total drain out, transfusion rates and with less of a drop in hemoglobin level. Another important finding of this meta-analysis was that topical TXA did not appear to increase the risk of DVT and PE compared with the placebo groups.

Tranexamic acid is a synthetic analog of the amino acid lysine that acts by blocking the lysine binding sites on plasminogen molecules, inhibiting the formation of plasmin [31]. Systematic review and meta-analysis has shown the efficacy of intravenous TXA on reducing blood loss and decrease the transfusion rates in THA [12]. However, concerns still exits regarding the safety of the systemic administration of TXA and the risk of thromboembolic events in the high-risk patient population undergoing joint replacements. Topical application of TXA during joint replacement may be a safer route of administration that will produce the same efficacy, but with much lower systemic absorption and thus much lower risk for thromboembolic complications [32].

The benefit of of topical application of tranexamic acid included: (1) direct targeting of the surgical site with a maximum concentration of TXA; and (2) prevention of systemic side effects. Recent meta-analyses has been published supporting the use of intravenous TXA to reduce postoperative blood loss in TKA [33]; however, evidence on topical TXA use in THA were still deficient. Therefore, we identified all available studies comparing topical TXA with placebo to investigate the its efficacy and safety after THA.

The primary endpoints of our study were total blood loss, drain loss, and transfusion rates. Our results revealed that topical TXA significantly reduced total blood loss (MD = −297.65, 95 % CI −371.68, 116.08; *P* < 0.01), total drain out (MD = −164.68, 95 % CI −236.63, −92.73; *P* < 0.01), transfusion rates (OR = 0.26, 95 % CI 0.17, 0.40; *P* < 0.01) and with less of a drop in haemoglobin level (SMD = −0.66, 95 % CI −0.91, −0.41; *P* < 0.01). Other studies in the literature have reached similar conclusions. In a double-blind, randomized controlled trial of 161 patients undergoing THA, Alshryda et al. [22] investigated the effect of topical application of TXA on blood loss and found that topical TXA was effective in reducing blood loss and the need for blood transfusion. Yue et al. [21] also conducted a randomized double blind controlled trial to assess the effect of topical TXA in THA and their results demonstrated that 3 g topical TXA could significantly reduce blood loss, drain blood loss, transfusions rates and with less of a drop in haemoglobin level.

In addition, one issue that must be addressed is whether topical TXA increases the risk of complications including DVT and PE. Of all 14 included studies, six studies reported the incidence of DVT or PE after THA. Total, five cases of DVT (1.4 %) was found in topical TXA group and eight cases was in placebo groups (0.6 %), which did not reach a significant statistical difference. Recently, Alshryda et al. [33] conducted a meta-analysis investigating the safety of topical TXA and indicated that there was no superiority in the rate of thromboembolic events in TXA group compared to control group in this study. Besides, our study did not show any heterogeneity in DVT among studies although different ways of prophylaxis in included studies.

Subgroup analysis based on the study type showed no statistically significant difference in main outcomes. In this meta-analysis, significant heterogeneity was observed in some outcomes. Regarding the methodological quality of included studies, seven studies were classified as A, six studies was B while one study was C. Besides, clinical heterogeneity was acknowledged by the following factors: first, clinical heterogeneity may be caused by blood transfusion protocol and different surgical approach (anterior, posterior or lateral). Second, with regards to the TA dose, different regimens were used by included studies ranging from 0.5 to 3 g, in 5 to 100 ml of Saline. However, subgroup analysis was not performed for TXA dose for lack of available data. To clarify this issue, further studies with a larger sample size are required to figure out which dose is better in topical application.

### Strength and limitation

There were several strengths of our review and metaanalysis. First, we performed exhaustive searches of all available studies, ncluding publications in any language as well as unpublished abstracts. Second, this meta-analysis was conducted strictly according to the methods of the Cochrane Handbook for Systematic Reviews of Interventions and PRISMA statement, ensuring the methodological or reporting quality of this meta-analysis.

The findings of this study need to be interpreted with considerations of the following limitations. Firstly, to identify all available studies, both RCTs and nRCTs were included for our analysis, which may decrease the level of evidence of our studies. Secondly, there was significant heterogeneity among main outcomes. Variations in methodology and different dose of TXA among the trials may have accounted for such heterogeneity. In order to eliminate heterogeneity, subgroup analysis was performed based on study type. Besides, insufficient data are available to support our intention to analyze functional outcome or quality of life measurement.

## Conclusions

This meta-analysis of the current literature indicates that topical TXA administration leads to a statistically significant reduction in total blood loss, drainage loss, transfusion rate and with less of a drop in haemoglobin level, with no increased risk of thromboembolic complications. Further study is required to elucidate the clinical outcomes and the optimal dose of topical TXA use in THA.

## Abbreviations

THA: total hip arthroplasty
TXA: tranexamic acid
